# The effects of a national, voluntary agreement for a more inclusive working life on work participation following long-term sickness absence: a Norwegian cohort study

**DOI:** 10.5271/sjweh.4112

**Published:** 2023-10-01

**Authors:** Rachel L Hasting, Ingrid S Mehlum, Karina Undem, Suzan JW Robroek, Alex Burdorf, Jon Michael Gran, Suzanne L Merkus

**Affiliations:** 1 Research Group for Occupational Medicine and Epidemiology, National Institute of Occupational Health, Oslo, Norway.; 2 Department of Community Medicine and Global Health, Institute of Health and Society, University of Oslo, Oslo, Norway.; 3 Erasmus University Medical Center Rotterdam, Department of Public Health, Rotterdam, The Netherlands.; ^4 ^Oslo Centre for Biostatistics and Epidemiology, Oslo University Hospital, Oslo, Norway.; 5 Oslo Centre for Biostatistics and Epidemiology, Department of Biostatistics, University of Oslo, Oslo, Norway.; 6 Research Group for Work Psychology and Physiology, National Institute of Occupational Health, Oslo, Norway.

**Keywords:** absenteeism, gender, longitudinal study, musculoskeletal, non-employment, Norway, psychological diagnose, return-to-work, sick leave

## Abstract

**Objectives:**

This study aimed to estimate the average individual effect of the company-level Norwegian Agreement on a More Inclusive Working Life (IA Agreement) on individuals’ (i) sustained return to work after a sickness absence (SA) episode, and (ii) recurrent SA.

**Methods:**

Using register data, 79 253 men and 94 914 women born in Norway 1967–1976 were followed for one year between 2005 and 2010 after returning to work from an SA episode (>16 days). Weighted Cox proportional hazard models analysed time to first exit from work by companies’ IA status (IA/non-IA). Weighted cumulative incidence differences between IA and non-IA groups with 95% bootstrapped confidence intervals (CI) were calculated for the competing events of full SA, graded (<100%) SA, unemployment/economic inactivity, education, disability pension, and death/emigration. Stabilised inverse probability of treatment weights balanced IA/non-IA groups according to nine covariates. Analyses were stratified by gender, and separately for two initial SA diagnoses (musculoskeletal and psychological).

**Results:**

Both men [adjusted hazard ratio (HR) 0.96, 95% CI 0.93–0.99] and women (adjusted HR 0.97, 95% CI 0.94–0.99) in IA companies were less likely to exit work in the year following SA. Similar findings were seen among individuals with musculoskeletal diagnoses and women with psychological diagnoses. Men with psychological diagnoses were more likely to exit work. Recurrent full and graded SA were more likely, and unemployment/economic inactivity less likely, in IA companies. However, the estimated effects were small and the CI often included the null.

**Conclusions:**

Individuals working in IA companies were more likely to remain in work. This was mainly due to reduced unemployment/economic inactivity, suggesting the IA Agreement may have influenced work participation through other means than reduced SA.

Absence due to illness and loss of paid employment have a negative impact both at the societal and the individual level. European Union (EU) member states spent approximately 1.1% of their GDP on sickness absence (SA) benefits in 2019 (1). In the EU an average of 12.4 days per worker were lost due to SA in 2018 (2). In Norway, the corresponding number was 16 days in 2019, equivalent to 5.9% of available work days (2, 3). Recurrent SA episodes can increase the risk of individuals’ permanent exclusion from working life and lead to financial issues and poor mental health, particularly if experienced early on in working life (4, 5). Therefore, reducing SA can have positive and long-lasting effects.

In 2001, the Norwegian Government and organisations representing employers and employees committed to increasing work participation through the national Agreement on a More Inclusive Working Life (hereafter the IA Agreement) (6). The IA Agreement had three aims, to: (i) reduce SA by 20% from its 2001 level (around 7%); (ii) include more individuals in the labor market and prevent withdrawal; and (iii) increase the pension age. Companies could sign the IA Agreement voluntarily, becoming “IA companies” who received tailored help from Working Life Centres administered by the Norwegian Labor and Welfare Administration (NAV). This included help with grants for workplace adjustments and a contact person for IA-related queries (see figure 1). The IA Agreement has been renewed several times, most recently up to 2024 (7), and was expanded in 2019 to cover all companies in Norway (8).

**Figure 1:**
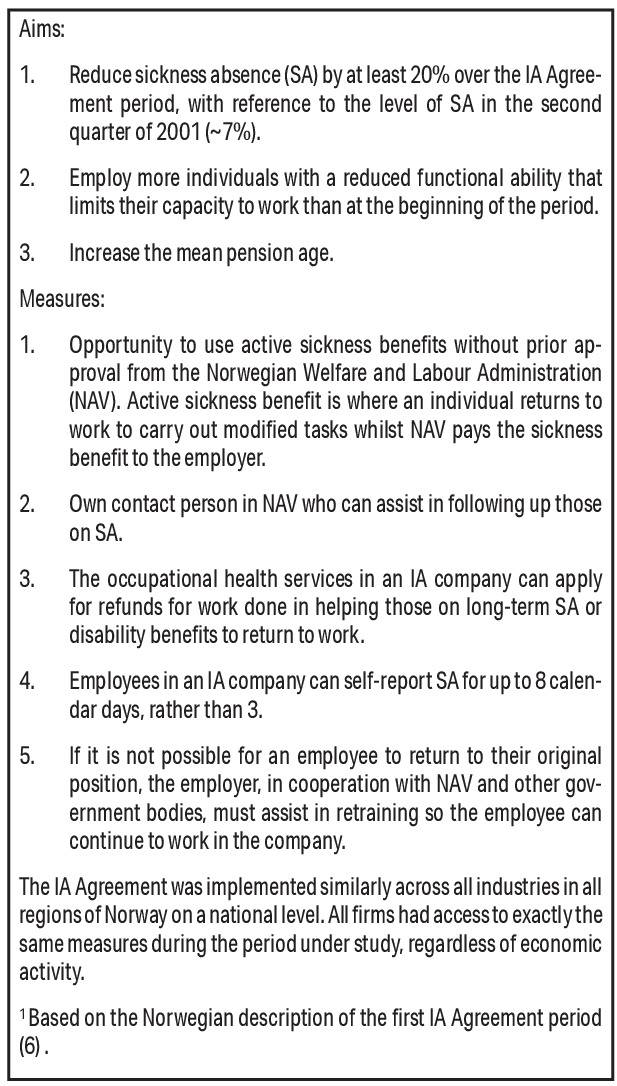
Description of the original Agreement on a More Inclusive Working Life (IA Agreement) goals and specific measures; adapted from Hasting et al (33).

The largest focus of the IA Agreement has been on reducing SA. However, between 2001 and 2018, SA has only been reduced by 12.4%, short of the 20% goal (9). Possible effects of the IA Agreement on SA have been investigated in several studies, with results varying from no effect to a possible positive effect of reducing SA prevalence and duration (10–14). A recent report indicated that recurrent SA may contribute to difficulties in reducing the overall SA rate, leading to a renewed focus on this in the current IA Agreement (8, 15). No studies have focused specifically on whether the IA Agreement has affected recurrent SA.

As in most countries, Norway has gender differences in SA, with women lying around three percentage points higher than men in physician-certified SA (15, 16). This gap is largest in adults aged 30–34 years (17) and is only partly due to pregnancy-related SA and having children (15, 18). Musculoskeletal and psychological diagnoses are the two largest causes of SA, responsible for 30% and 20% of days lost, respectively (15). They are also associated with a high degree of recurrent SA and exit from paid employment (19). Men are more likely to have musculoskeletal-related SA, whilst women are more likely to have psychological-related SA (15). Previous studies suggest that both gender and diagnosis group may respond differently to the tools used in the IA Agreement (10, 20, 21), and would benefit from being studied separately.

The second goal of the IA Agreement is associated with the inclusion of individuals who are naturally more prone to SA, thus increasing the SA rate (22). This goal is therefore in direct conflict with the goal to reduce SA and suggests a more holistic approach should be considered when evaluating the effects of the IA Agreement. The aim of this study was to assess the effect of the IA Agreement (signed at a company-level) on individuals’ remaining in work and risk of recurrent SA, following an initial SA episode among young to middle-aged adults. Men and women were studied separately, and a particular focus was on those returning from musculoskeletal and psychological SA.

## Methods

### Data sources

This study utilized a Norwegian cohort comprised of all individuals live-born in Norway between 1967 and 1976 (N=626 928), linking registries using the unique individual identification number. The “FD-Trygd” events database (23), maintained by Statistics Norway (SSB), was used for the following: employment dates, SA (>16 calendar days) dates and grade, SA follow-on benefits (medical and vocational rehabilitation/work assessment allowance) dates and grade, unemployment dates, disability retirement date, death date, emigration date, company industry, and company region. In Norway, SA episodes are registered in the database when the responsibility for covering the benefits passes from the employer to NAV after 16 calendar days, so only episodes longer than this were included. Data on birth year and month, gender, and civil status were obtained from SSB, and are based on the National Population Register (24, 25). Education information came from the National Education Database (NUDB), maintained by SSB (26). Information on company size (number of employees) came from the Central Register of Establishments and Enterprises, maintained by SSB (27). Data on if/when companies signed the IA Agreement, any changes to their agreement status, and SA diagnoses were obtained from NAV. Ethical approval was obtained from the Regional Committee for Medical and Health Research Ethics (case number 17344).

### Study design and population

**Figure 2:**
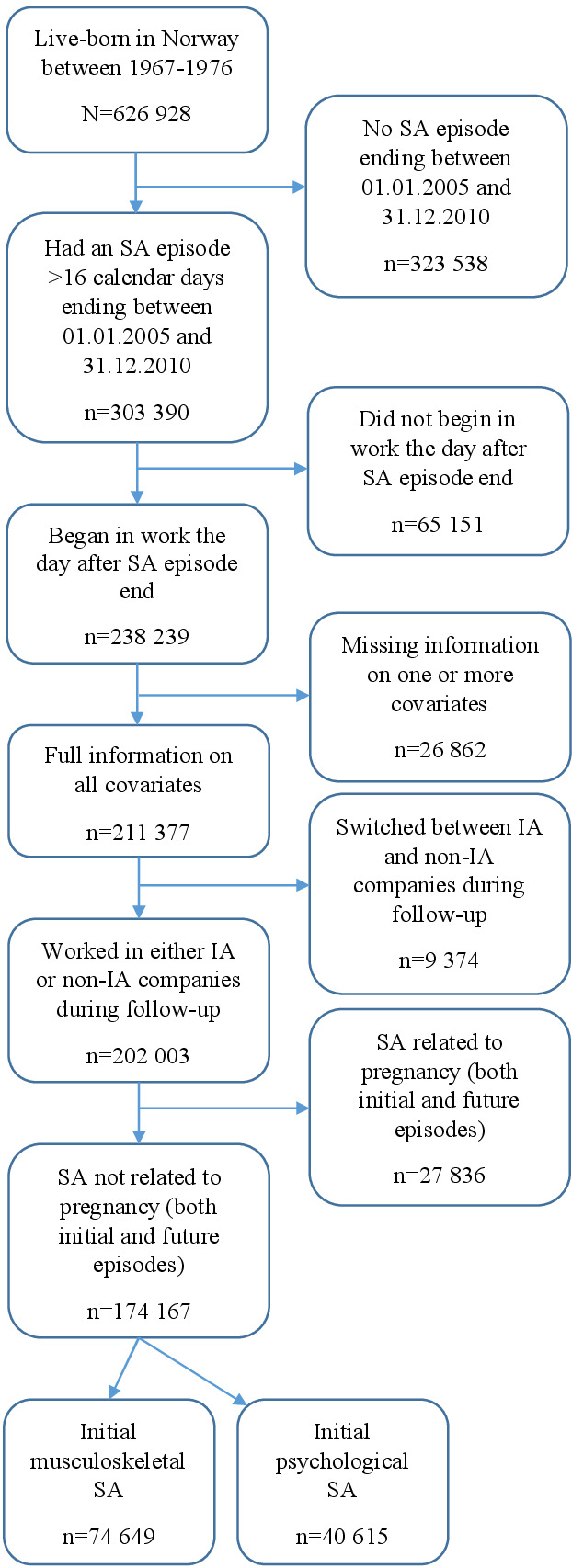
Population flowchart.

This cohort study included individuals the day after their first SA episode ended between 1 January 2005 and 31 December 2010 (t=0, ie, the first day with no SA). The source population consisted of 303 390 individuals aged 28–37 years on 1 January 2005 (figure 2). To be included in this study, individuals were required to start work the day following the end of their SA episode (N=238 239) and to have full information for all covariates (N=211 377). To ensure the intervention was well-defined, individuals were included if they had only worked in either IA or non-IA companies during their 1-year follow-up period (N=202 003). For the purposes of ensuring the SA of men and women were comparable, those returning from pregnancy-related diagnoses were excluded. All individuals were followed for 1 year (until t=364). Thus, the study period was 2005–2011.

### Study outcomes

We used the risk of exiting work due to any cause as our first outcome. This included both temporary and permanent lapses in work participation. To assess the risk of recurrent SA, we analyzed the risk of full and graded SA in the presence of other competing events. We defined SA as receiving SA benefits, which individuals have a right to for up to 52 weeks, as well as follow-on benefits (rehabilitation until 2010, work assessment allowance after 2010), which can be applied for after the right to paid SA has ceased and can last for a further five years (28). We did not have data on grade for work assessment allowance, so this was categorized as full SA. Parental leave and annual leave were included in work.

It was possible for individuals to experience more than one event simultaneously; therefore, we used a hierarchy to prioritize the most important events for our study objectives, given the other competing events. This resulted in the following prioritization:

1. Full SA (100%)
2. Graded SA (<100%)
3. Unemployment/economic inactivity
4. Education
5. Disability pension
6. Death and emigration

Individuals were censored if they were still in work at the end of the 1-year follow-up. If individuals had a gap of <2 months between two jobs and did not experience another event in this time, the gap was considered work; if the gap was >2 months, the individual was classified in “unemployment/economic inactivity”.

### Intervention: IA Agreement

We aimed to identify the average effect of having access to the IA Agreement compared to not having access to the IA Agreement at the time of first return to work after sick leave in the period 2005–2011. “Having access” was defined as working at baseline in a company that had signed the IA Agreement (an “IA company”). Employees working in IA companies were compared to employees working in non-IA companies, adjusting for baseline differences between groups. IA status was coded as a binary variable (yes/no) and was recorded annually.

### Covariates

Covariates included in this study were calendar year (at baseline), age (in years), civil status, education level, length of initial SA (in days), grade of initial SA, industry, company size, and company region. All covariates were measured at baseline (t=0). Civil status was coded into a binary variable denoting single or married/in a civil partnership. Education was coded into five categories based on the Norwegian Standard Classification of Education (NUS2000) (29): lower secondary education or lower, upper secondary (basic), upper secondary (completed), tertiary (undergraduate), and tertiary (graduate). Grade of initial SA was included as a binary variable denoting full (100%) or partial (<100%) sick leave. The industry variable was coded according to the Standard Industrial Classification 2002 (30), based on either the Statistical Classification of Economic Activities in the European Community (NACE) Revision 1.1 before 2009, or NACE Revision 2 after 2009, and included 13 different industrial categories (see table 1). Company size was measured by number of employees and was modelled using a linear spline with three knots. Company region was coded into east, south, west, middle, or north. Where possible, missing values were imputed from either the previous year or the following year; otherwise, the individual was excluded from analysis.

**Table 1:** Characteristics of the main study population (N=174 167), stratified by gender and Agreement on a More Inclusive Working Life (IA) status at baseline. All covariates are measured at baseline (t=0). [SA=sickness absence; ICPC= International Classification of Primary Care.]

		Men (N=79 253)						Women (N=94 914)				
		IA (N=29 697)			Non-IA (N=49 556)			IA (N=51 930)			Non-IA (N=42 984)	
		Quartiles	N (%)		Quartiles	N (%)		Quartiles	N (%)		Quartiles	N (%)
Age (years)		34 – 36 - 38			33 – 36 - 38			34 – 36 - 38			33 – 35 - 38	
Education												
	Lower secondary or below		4 069 (14)			10 727 (22)			5394 (10)			7844 (18)
	Upper secondary, basic		1709 (6)			4050 (8)			3571 (7)			3721 (9)
	Upper secondary, completed		13 552 (46)			26 105 (53)			15 432 (30)			17 506 (41)
	Tertiary, undergraduate		7895 (27)			6940 (14)			23 495 (45)			12 005 (28)
	Tertiary, graduate		2472 (8)			1734 (4)			4038 (8)			1908 (4)
Civil status												
	Single		17 762 (60)			31 337 (63)			27 872 (54)			24 507 (57)
	Married/in a civil partnership		11 935 (40)			18 219 (37)			24 058 (46)			18 477 (43)
Industry												
	Agriculture/forestry/fishing		139 (<1)			1041 (2)			57 (<1)			373 (1)
	Mining/quarrying		465 (2)			1602 (3)			294 (1)			288 (1)
	Manufacturing		7377 (25)			8421 (17)			2865 (6)			3214 (7)
	Electricity/gas/water supply		316 (1)			217 (<1)			166 (<1)			95 (<1)
	Construction		3016 (10)			8966 (18)			318 (1)			629 (1)
	Wholesale/retail		2176 (7)			10 384 (21)			2272 (4)			11 447 (27)
	Hotels/restaurants		294 (1)			1012 (2)			773 (1)			1939 (5)
	Transport/storage		2529 (9)			6242 (13)			1789 (3)			2300 (5)
	Financial/real estate		2077 (7)			6505 (13)			2768 (5)			6 893 (16)
	Public administration		2873 (10)			961 (2)			4173 (8)			1292 (3)
	Education		3069 (10)			578 (1)			9 031 (17)			1678 (4)
	Health/social		4358 (15)			1657 (3)			26 189 (50)			10 161 (24)
	Other		1008 (3)			1970 (4)			1235 (2)			2675 (6)
Number of employees		38 – 100 – 320			8 – 22 – 68			27 – 69 – 213			8 – 19 – 57	
Work region in Norway												
	East		13 749 (46)			22 905 (46)			22 954 (44)			21 874 (51)
	South		2863 (10)			4077 (8)			4489 (9)			3457 (8)
	West		7295 (25)			11 747 (24)			13 256 (26)			9 575 (22)
	Middle		2 676 (9)			4578 (9)			5008 (10)			3867 (9)
	North		3114 (10)			6 249 (13)			6223 (12)			4 211 (10)
Initial SA diagnosis												
	Musculoskeletal (ICPC code L)		14 013 (47)			24 973 (50)			18 968 (37)			16 695 (39)
	Psychological (ICPC code P)		6162 (21)			9540 (19)			13 794 (27)			11 119 (26)
	Other		9522 (32)			15 043 (30)			19 168 (37)			15 170 (35)
Grade of initial SA												
	Full (100%)		22 546 (76)			38 194 (77)			32 772 (63)			27 365 (64)
	Partial (<100%)		7151 (24)			11 362 (23)			19 158 (37)			15 619 (36)

### Statistical analyses

The data we had were purely observational and registry-based. In order to come closer to an RCT, we used methods from the causal inference field that allowed us to better emulate randomization and give a more causal interpretation of the results. This is valid given that certain conditions hold; consistency (the IA Agreement is well-defined), exchangeability (those in the non-IA group would have had the same average outcome as those in the IA group had they also been in the IA group), and positivity (at least one individual in the IA and non-IA groups have every combination of the covariates) (31).

As the IA Agreement is voluntary, IA companies and their employees are likely to differ from non-IA companies and their employees. To adjust for such differences, stabilized inverse probability of treatment weights (sIPTW) were calculated using logistic regression and used to weight individuals based on their probability of having an IA Agreement according to their combination of the nine covariates described above. Analyses were then performed on the weighted dataset, where the two groups (IA/non-IA) can be considered balanced with respect to the covariates.

To analyze the probability of exit from work due to any cause, weighted gender-specific Cox proportional hazard models were used to analyze time to exit from work by IA status.

To analyze recurrent SA, we first calculated weighted cumulative incidence curves for all individual causes of exit from work (described above) plus the likelihood of remaining in work. This method considers competing risks from other outcomes than SA. We used the “stcompet” command in Stata. The gender-specific analyses were stratified by IA status. For each competing event, the absolute difference in cumulative incidence between IA and non-IA groups were visualized in graphs along with 95% confidence intervals (CI) generated by clustered bootstrapping with resampling conducted at a company level (1000 repetitions).

The same analyses were performed separately on those returning from SA with a musculoskeletal diagnosis and a psychological diagnosis, respectively. The diagnoses were identified using the International Classification of Primary Care (ICPC-2) codes for diagnoses (L for musculoskeletal diagnoses, and P for psychological diagnoses) (32).

All analyses were conducted in Stata, version 16.1 (StataCorp, College Station, TX, USA).

## Results

The final study population was comprised of 174 167 individuals (57% of the source population, figure 1). Total follow-up time was 49 632 881 days (135 887 years), with an average follow-up time of 285 (standard deviation (SD) 118) days. In the IA population, the average follow-up time was 286 (SD 119) days, whilst in the non-IA population it was 284 (SD 118) days.

Table 1 shows the population characteristics. Men worked more often in non-IA companies and women more often in IA companies. Men and women working in IA companies tended to have a higher education level. The majority of IA companies were in the manufacturing (for men), health/social (particularly for women), education and public administration industries. IA companies also had on average a higher number of employees than non-IA companies. Finally, for diagnosis-specific analyses, IA companies had slightly fewer individuals returning from a musculoskeletal-related SA and slightly more returning from a psychological-related SA than non-IA companies.

### Effect of the IA Agreement on remaining in work

**Figure 3:**
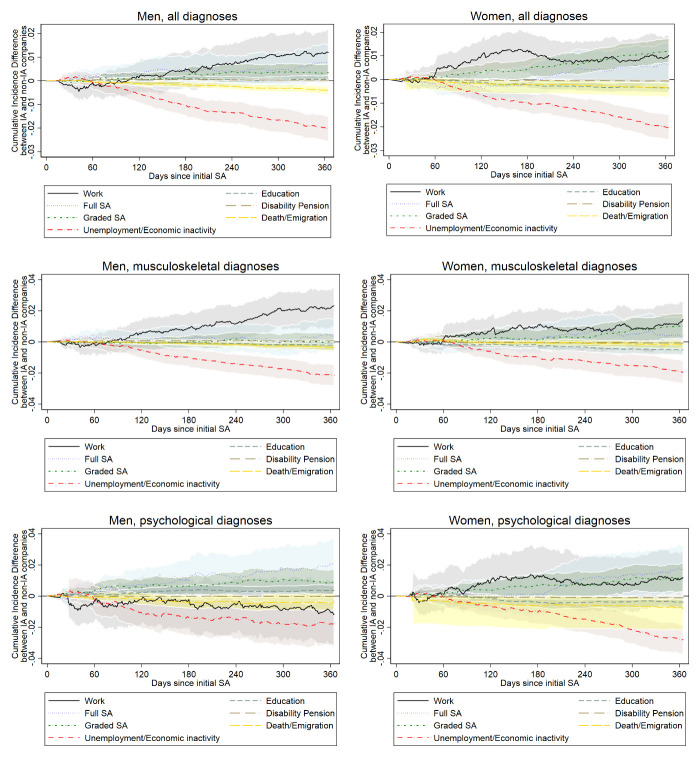
Difference in cumulative incidence for employees in Agreement on a More Inclusive Working Life (IA) companies compared to those in non-IA companies, for the following states: work, full sickness absence (SA), graded SA, unemployment/economic activity, education, disability pension, and death/emigration. Stratified by gender and diagnosis. 95% confidence intervals calculated using 1000 bootstrap samples.

Over half of the weighted study population remained in work following SA (table 2). Compared to non-IA companies, both men and women working in IA companies were more likely to remain in work throughout the 1-year follow-up, ie, they had a lower risk of all-cause exit from work [hazard ratio (HR) men: 0.96, 95% CI 0.93–0.99; HR women: 0.97, 95% CI 0.94–0.99] (table 3). This estimated effect was seen after 100 days for men, and after 60 days for women (figure 3).

**Table 2:** Number of individuals remaining in work and events experienced during follow-up for the weighted study population (N=172 769), stratified by gender and Agreement on a More Inclusive Working Life (IA) status at baseline. All covariates are measured at baseline (t=0). [SA=sickness absence.]

	Men (N=78 489)				Women (N=94 280)		
	IA (N=28 878)		Non-IA (N=49 611)		IA (N=52 309)		Non-IA (N=41 971)
	N (%)		N (%)		N (%)		N (%)
Remained in work	19 175 (66)		32 346 (65)		30 302 (58)		23 887 (57)
Full (100%) SA	5645 (20)		9319 (19)		11 302 (22)		8797 (21)
Graded (<100%) SA	1434 (5)		2293 (5)		5444 (10)		3865 (9)
Unemployment/economic inactivity	2074 (7)		4568 (9)		4130 (8)		4166 (10)
Education	238 (1)		374 (1)		617 (1)		645 (2)
Disability pension	31 (<1)		39 (<1)		57 (<1)		72 (<1)
Death/emigration	281 (1)		672 (1)		457 (1)		539 (1)

**Table 3:** Weighted ^a^ Cox proportional hazard models for the risk of all-cause exit from work after an initial sickness absence (SA) episode during a 1-year follow-up between 2005 and 2011 in Agreement on a More Inclusive Working Life (IA) companies compared to non-IA companies, stratified by gender and initial SA diagnosis group. [HR=hazard ratio; CI=confidence interval.]

		All-cause exit in IA companies vs non-IA companies
		HR (95% CI)
Men		
	All-cause initial sickness absence (N=79 253)	0.96 (0.93–0.99)
	Musculoskeletal (N=38 986)	0.92 (0.88–0.97)
	Psychological (N=15 702)	1.04 (0.97–1.11)
Women		
	All-cause initial sickness absence (N=94 914)	0.97 (0.94–0.99)
	Musculoskeletal (N=35 663)	0.96 (0.92–1.00)
	Psychological (N=24 913)	0.96 (0.91–1.02)

^a^ All analyses were weighted for calendar year (at baseline), age, civil status, education level, length of initial SA (days), grade of initial SA, industry, company size, and company region.

### Effect of the IA Agreement on recurrent SA

Approximately a quarter to a third of the weighted study population experienced recurrent SA (table 2). The differences between IA and non-IA groups in cumulative incidence for recurrent SA ranged from approximately -0.2 to 1.2 percentage points by the end of the 1-year follow-up (figure 3). A negative value indicates the outcome is less likely to occur in IA companies compared to non-IA companies, whereas a positive value indicates the outcome is more likely to occur in IA companies. Individuals working in IA companies were more likely to have full SA during follow-up than those in non-IA companies, though this was not before 120 days for men and 180 days for women. Compared to non-IA companies, both men and women working in IA companies were more likely to have graded SA during follow-up. This difference was larger for women than for men and increased in both groups over time.

### Effect of the IA Agreement on other labor market exits

The differences in cumulative incidence for the other labor market exits from work ranged from approximately -2–0 percentage points by the end of the 1-year follow-up. Both men and women working in IA companies were less likely to experience unemployment/economic inactivity than those working in non-IA companies; this difference increased over time (figure 3).

### Effect of the IA Agreement on sustained return to work after SA due to musculoskeletal disorders

Following an SA episode with a musculoskeletal diagnosis, both men and women working in IA companies had a lower risk of all-cause exit from work than those working in non-IA companies (table 3). This higher likelihood of remaining in work is seen in the graph after 100 days for men and 60 days for women (figure 3).

There were no obvious differences in recurrent all-cause SA, either graded or full, following a musculoskeletal diagnosis in men (figure 3). Women were slightly more likely to experience graded SA following the first 100 days back in work, and full SA after 120 days, if they worked in an IA company compared to a non-IA company. Both genders were less likely to be unemployed/economically inactive if they were working in an IA company, compared to a non-IA company.

### Effect of the IA Agreement on sustained return to work after SA related to psychological disorders

Following an SA episode with a psychological diagnosis, men working in IA companies had a higher risk of all-cause exit from work (HR 1.04, 95% CI 0.97–1.11; table 3); they were less likely to remain in work throughout follow-up than men in non-IA companies (figure 3). They were also more likely to experience full or graded SA but less likely to experience unemployment/economic inactivity.

Women working in IA companies were more likely to remain in work when returning from an SA episode with a psychological diagnosis than women in non-IA companies (HR 0.96, 95% CI 0.91–1.02; table 3); this was seen after the first 50 days (figure 3). Women were more likely to experience graded SA throughout follow-up if they worked in an IA company, as well as full SA after 200 days in work, and less likely to be unemployed/economically inactive compared with those working in non-IA companies.

## Discussion

Our results indicate that both men and women were somewhat more likely to remain in work in the year following SA (all-cause or musculoskeletal) if they worked in a company that had signed the IA Agreement. There was a gender difference among those returning from psychological SA. Compared to those in a non-IA company, men were less likely to remain in work while women were more likely to remain in work if they worked in an IA company. Both men and women in IA companies were slightly more likely to have recurrent full and graded SA, including when returning from musculoskeletal or psychological SA. However, the estimated effects for psychological SA and for recurrent SA were small and the CI often included the null.

These findings suggest that the IA Agreement is successfully increasing work participation, albeit to a small extent. Individuals working in a company that had signed the IA Agreement were on average more likely to remain in work (except men returning from a psychological SA) and less likely to end up unemployed/economically inactive following an SA episode. This indicates that IA companies prevent withdrawal of potentially sicker individuals to a greater extent than non-IA companies, as the second goal of the IA Agreement promotes. The estimated effect size was small, but when extrapolated to the larger working population this can translate to many working days that would otherwise have been lost. The variation in estimated effect sizes suggests that there may be differences in how the IA Agreement affects different genders and diagnosis groups.

We found that individuals working in IA companies were more likely to remain in work but also more likely to experience recurrent SA compared to those in non-IA companies. To our knowledge, this is the first study that has specifically aimed to estimate the effect of the IA Agreement on recurrent SA. We cannot therefore directly compare our findings to previous studies. More general research on SA and the IA Agreement indicates that IA companies have generally higher rates of SA, in line with our findings (12, 33, 34). The similarity between the present study findings and research into general SA could indicate that the mechanism by which the IA Agreement affects recurrent SA is not very different. Although IA companies may have higher rates of SA, previous studies suggest that the IA Agreement may have a small positive effect on SA duration (14, 33). If the IA Agreement works mainly by shortening SA duration, this may contribute to achieving the overall goal of reducing SA by reducing working days lost, rather than reducing the occurrence of SA (8).

The higher likelihood of remaining in work in this study was most likely related to the lower risk of being unemployed/economically inactive. The simultaneous increase in SA and decrease in unemployment/economic inactivity suggests that individuals who would otherwise have stopped working for health-related reasons may instead have more recurrent SA. Additionally, it is possible that IA companies are better at including individuals with reduced work capacity, which is in line with the second IA Agreement goal. This would also increase SA whilst reducing more permanent labor market exits such as unemployment/economic inactivity. These results also support the concept of “communicating vessels”, where reducing one exit method results in increased exit by another route (35). A recent study into those beginning SA episodes between 2004–2011 in the same cohort similarly found a decreased risk of non-employment (not in work, education, or SA) in IA companies (14). The differences between IA and non-IA companies appear after around 60 days, suggesting that the measures may work effectively for diagnoses where adjustments reduce the risk of relapse over time.

An interesting finding was that men returning from psychological SA seem to be less likely to remain in work and more likely to experience recurrent SA if they worked in IA companies compared to non-IA companies. This contrasts with the other analysis groups and may be because psychological conditions tend to be underdiagnosed or diagnosed later in men, who are less likely than women to visit their doctor with health concerns (36, 37). If IA companies are better at keeping people in work, as this study suggests, it may be the case that men who return from psychological SA and work in IA companies have more serious conditions than those in non-IA companies. Only one other study has looked specifically at the IA Agreement and psychological diagnoses among men, finding a slight decrease in initial SA prevalence and duration in IA companies (33). The differing results here may be because the present study looks at recurrent SA specifically, rather than SA more generally.

### Methodological considerations

The strengths of this study include the use of register-based data, which are assumed to be complete without loss to follow-up and are collected objectively. The methods used took into account competing risks, presenting a more accurate picture of the IA Agreement’s effects on recurrent SA than other observational methods and setting it in a larger context (38). Due to its voluntary nature, it was not possible to evaluate the IA Agreement using experimental methods, which would be the ideal for our aim of estimating the average effect. We have therefore used methods from the field of causal inference, such as sIPTW weighting, to ensure the groups are balanced with respect to confounding factors and come closer to the randomization that is possible in experimental studies (31). This increases the generalizability of our findings to others in a similar age range and enables a causal interpretation of our results given the criteria of consistency, exchangeability, and positivity, which we believe to be satisfied in our study (31). However, there is always a possibility of residual confounding, which means our estimated effect may differ from the true effect. The population in this study is restricted to young and middle-aged adults (28–44 years), which means the results cannot be generalized to older workers, who may have a different pattern of SA or who may exit work faster/more often than younger workers (18, 20).

A weakness of our study is that we only had information on whether the companies had signed the IA Agreement, not on their actual use of IA measures, which can vary greatly between IA companies (20). Additionally, we did not have information on all covariates that likely influence whether companies sign the IA Agreement, such as whether they belong to the public or private sector (39). We have, however, adjusted for other company-level covariates such as industry, which can account for some of this potential confounding.

We did not have information on SA episodes <17 calendar days. If the IA Agreement does reduce SA duration (33), some individuals in IA companies would not be included because their SA would be <17 days. This would lead to selection bias, where IA individuals returning from SA in our study may be sicker and more prone to recurrent SA than non-IA individuals, which could explain the higher rate of recurrent SA found in this study. This may result in an underestimation of the effects of the IA Agreement on recurrent SA. We included duration and grade of initial SA in the IPTW weights to account for these sources of bias, but it is possible that some bias still remains.

### Implications and future research

The small estimated effect sizes observed in this study can be meaningful on the larger scale. After all-cause SA, men in IA companies had an average of 297 days until exit from work during the 1-year follow-up period, whereas for non-IA companies this was 291 days (6 days’ difference; data not shown). For women, the corresponding numbers were 280 days for those in IA companies and 276 days for those in non-IA companies (4 days’ difference). As around 5% of employed men and 10% of employed women aged between 30–44 had physician-certified SA at the end of 2021 (40), these differences would amount to a considerable number of working days gained over the course of a year.

There is a need to better understand the effects of graded SA and to what extent this may substitute full SA. Additional research into the duration and frequency of all SA episodes during an individual’s working life would aid assessments of to what extent the IA Agreement is reaching its goals. Explanations for why men with psychological diagnoses may have different outcomes to the other groups should be further investigated.

Finally, it would be interesting to look at effects of the IA Agreement on work participation in a more general working population, ie, not only individuals returning from SA, and see if this affects the pattern observed in this study. Studying multiple exits from work is beneficial for understanding the larger picture of the IA Agreement, as demonstrated by the findings in this study.

### Concluding remarks

In the year following an initial SA episode, access to the IA Agreement slightly increased the likelihood of sustained return to work and recurrent SA in men and women overall, and in those with musculoskeletal diagnoses. This may have been due to reducing withdrawal from work through unemployment/economic inactivity. Men with psychological diagnoses had a slightly lower likelihood of remaining in work if they worked in IA companies, which may be due to a higher risk of recurrent SA. The results of this study indicate that the IA Agreement is contributing to increasing participation in working life following an SA episode, but not necessarily through its goal of reducing SA.
